# *In silico* analysis of protein/peptide-based inhalers against SARS-CoV-2

**DOI:** 10.2217/fvl-2020-0119

**Published:** 2020-10-08

**Authors:** Saad Salman, Fahad Hassan Shah, Maham Chaudhry, Muniba Tariq, Muhammad Yasir Akbar, Muhammad Adnan

**Affiliations:** 1 ^1^Department of Pharmacy, The University of Lahore, Islamabad Campus, Islamabad, Federal 44000, Pakistan; 2 ^2^Centre of Biotechnology & Microbiology, University of Peshawar, Khyber Pakhtunkhwa, Pakistan; 3 ^3^Diet & Nutritional Sciences, The University of Lahore, Islamabad Campus, Islamabad, Federal 44000, Pakistan; 4 ^4^Department of Bioinformatics, Quaid-e-Azam University, Islamabad, Federal 44000, Pakistan

**Keywords:** COVID-19, dornase-alfa, inhalers, monoclonal antibody, short-palate-lung and nasal-epithelial clone-1-derived peptides

## Abstract

**Aim:** Peptide/protein-based inhalers are excessively used to treat respiratory disorders. The molecular docking was performed for these inhalers including human neutralizing S230 light chain-antibody (monoclonal antibodies [mAbs]), alpha-1-antitrypsin (AAT), short-palate-lung and nasal-epithelial clone-1-derived peptides (SPLUNC1) and dornase-alfa (DA) against spike glycoprotein of severe acute respiratory syndrome coronavirus 2 (SARS-CoV-2) to assess their inhibitory activity. **Materials & methods:** HawkDock was used to dock these biologics against SARS-CoV-2 spike-glycoprotein. **Results:** Results showed that DA, AAT and mAb were quite active against spike glycoprotein with a binding free energy of -26.35 and -22.94 kcal/mol. **Conclusion:** mAB and AAT combined with DA can be used in the treatment of coronavirus disease of 2019 as a potential anti-SARS-CoV-2 agent.

## Graphical abstract

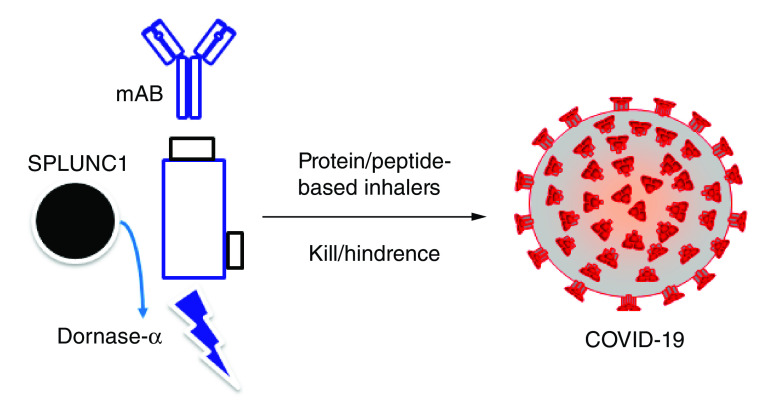

Coronavirus disease of 2019 (COVID-19) is an acute respiratory disorder that is similar to severe acute respiratory syndrome (SARS) and middle east respiratory syndrome (MERS) [1,2]. The origin and evolution of COVID-19 are of immense importance for the drug discovery and prevention of the epidemic. There are 28 proteins that encode by SARS-CoV-2, out of which, 16 are for replication, four are structural proteins responsible for viral packaging and pathogenesis while the remaining are the accessory proteins. The structural proteins are spike, membrane, nucleocapsid and envelope protein [3]. SARS-CoV-2 exploits crown-shaped spike glycoprotein to interact with the ACE-2 receptor to gain entry inside the host cell. The virus then reproduces to form abundant copies which on maturation burst open the infected cell and releases viral progenies which in turn attack other surrounding cells ultimately reaching the brain. SARS COV-2 spike glycoprotein consists of two subunits that are the S1 and S2. The S1 subunit consists of receptor binding domain (RBD) that binds with the host cell receptor to ACE-2. The second subunit is S2 which arbitrate fusion between viral and plasma membrane of host. It contains protrusions that allow the virus to bind a receptor on the host cell.

SARS-CoV-2 is transmitted by droplets and contact between a healthy individual and an infected person [4]. The spike glycoprotein is a 142.3 kDa protein present on the surface of the virion that facilitates interaction by employing their 319–591 number amino acid residues to interact with the ACE-2 receptor. After attachment, the infection develops, and the human body’s immediate response is to produce an immune response against the infection and also to modulate the mucous secretion in the surrounding epithelial cells to deter the attachment of other freshly produced SARS-CoV-2 progenies. Both SARS-CoV-2 and mucus aggravate disease progression by contributing to the production of a cough. It is imperative to develop aerosols that could help in the prevention and control of the disease.

Aerosols composed of monoclonal antibodies (mAbs) are commonly used for respiratory disorders, such as asthma [5]. mAbs are composed of transgenic animals or phage display technology, which benefit in several ways as to curtail immunogenicity, increase effector function and delay their serum half-life [6,7]. Cystic fibrosis (CF) airways manifest increased neutrophil elastase activity, which can likely destroy the lung, and also cleave and activate ENaC intensifying mucus dehydration and expediating mucociliary clearance. Alpha-1-antitrypsin (AAT) is an endogenous neutrophil elastase (NE) inhibitor that, by blocking NE, can improve pulmonary function [8]. The drug dornase-alfa (DA) for CF reduces DNA length, hydrolyzes DNA polymer and makes a good mucolytic agent. Making DA a drug of choice for CF that is well tolerated, helps in the amelioration of lung function and reduces sputum viscosity [7]. So, short-palate lung and nasal-epithelial clone-1-derived peptides (SPLUNC1) could be utilized in CF for the inhibition of ENaC [8] and regulation of Th2 inflammation [9]. SPX-101 is novel protease-resistant peptide that inhibits ENaC, regulates mucosal secretions, and ameliorates rehydration [10]. However, the use of protein/peptide-based inhalers does have toxicogenic and immunogenic responses in the body [11]. The administration of these agents could be debilitating, if the dose is not controlled or with their chronic use [12]. Some of these protein/peptide-based inhalers can precipitate pulmonary toxicity, cytokine-release syndrome, serum sickness, tumor lysis syndrome, toxic pulmonary-amyloid aggregates, antibodies generation and acute anaphylaxis, anaphylactoid reactions and in rare cases progressive multifocal leukoencephalopathy [13–15]. Although the safety and efficacy of DA [16], SPLUNC1 [17], AAT [18] and mAb [19] are well established, still the detailed studies regarding their toxic and antigenic profiles remain elusive. These four inhalers containing mAb, DA, AAT and SPLUNC1 were tested through molecular docking against SARS-CoV-2 spike glycoprotein, their toxicity, antigenicity, water solubility and resistance to gastric enzymatic activity were evaluated.

## Materials & methods

### Molecular docking

The protein ligands alpha-1-antitrypsin (PDB ID:1ATU), DA (human recombinant DNAse-I (PDB ID: 4AWN), angiotensin-converting enzyme-2 (ACE-2) (PDB ID:1R4L), human palate, lung and nasal epithelium clone protein (4N4X) and human neutralizing S230 light chain antibody (6NB6) and COVID spike protein (PDB ID:6VSB) was obtained from Protein Data Bank [20,21]. These protein structures were further refined with Modrefiner [22] and HawkDOCK [23] was then used for docking studies coupled with Discovery Studio 4.0 for the visualization of docked complexes.

### Toxicity assessment

**Figure 1.**
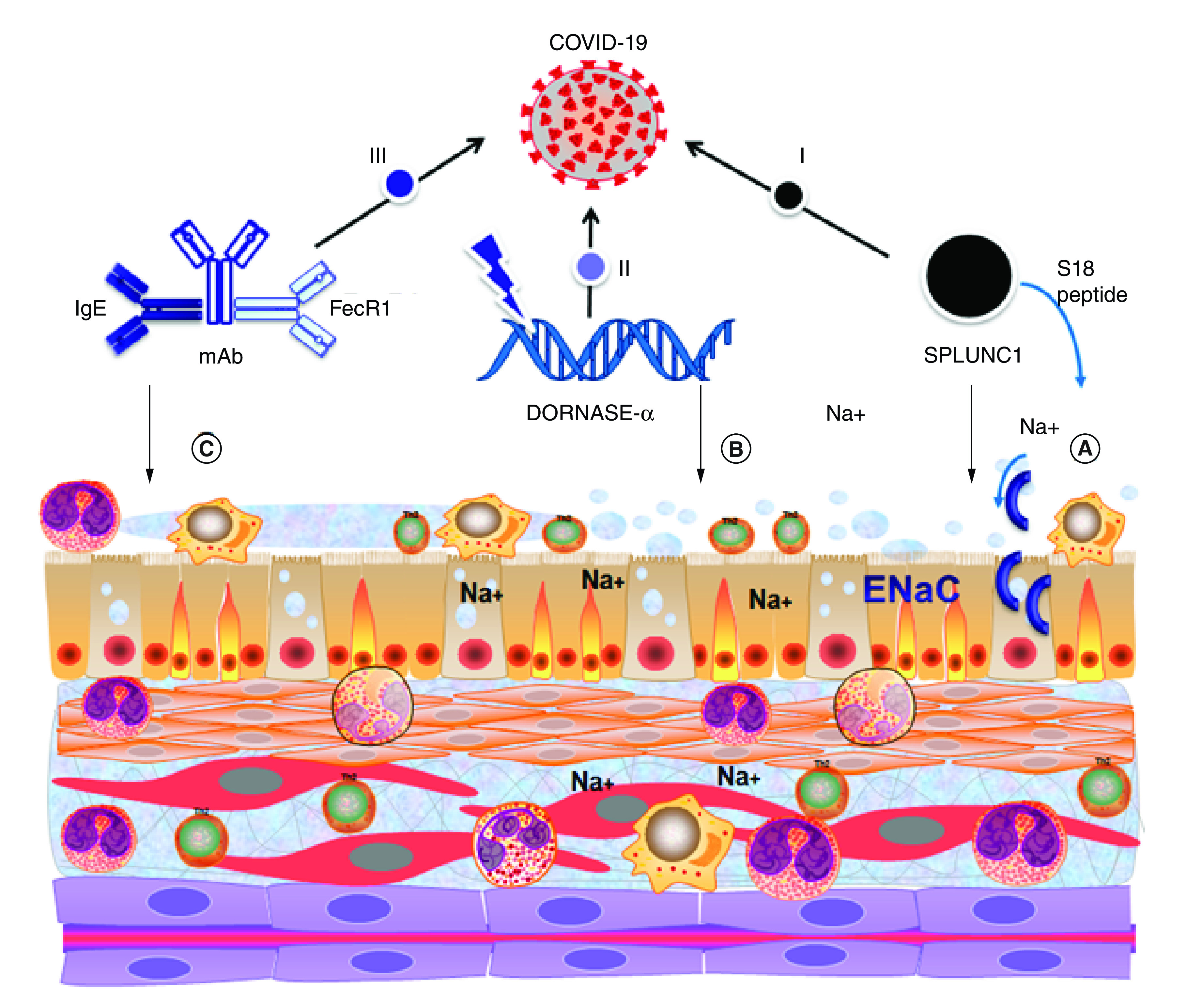
Pathophysiology of asthma and cystic fibrosis. **(A)** SPLUNC1 is a protein that consists of the ENaC inhibitory domain-S18 region. SPLUNC1-derived peptide, which serves in CF airways as an ENaC inhibitor, help in the regulation of lung function. **(B)** CF has high levels of DNA and actin in the lung lumen, which are discharged by necrotic neutrophils. Dnase1 cleaves extracellular DNA in the lung lumen reducing DNA length and sputum viscosity is decreased. mAbs get attached to a wide variety of proteins with high affinity and specificity. In asthma, they bind to IgE and FecRI resulting in reduced exacerbations. I–III: these three agents fight against SARS-CoV-2 by either binding (I, II) or blocking (III) its passage to reach out to the ACE-2 receptor. CF: Cystic fibrosis; mAb: Monoclonal antibody.

To assess the *in silico* toxicity analysis of the protein/peptide-based inhalers, the UniProt database was used for the protein/peptide sequences. The toxicity was analyzed by utilizing ToxinPred [24] while the prediction of allergenicity was calculated by utilizing AllerTop [24]. Also, these samples were subjected to *in silico* prediction of gastrointestinal digestion resistance by utilizing PeptideCutter [25]. The water solubility of these inhalers was checked through peptide property calculator, in order to establish its water solubility. The proposed mechanism of DA, SPLUNC1 and mAb in asthma and CF are given in Figure 1.

## Results

### Docking analysis

Protein–protein interaction (PPI) of COVID-19 spike glycoprotein with alpha-1-antitrypsin (1atu), dornase-alfa (4AWN), angiotensin-converting enzyme-2 (ACE-2) (PDB ID:1R4L), human palate, lung and nasal epithelium clone protein (SPLUNC1) (4n4x) and human neutralizing the S230 light chain antibody was evaluated through HawkDock. The algorithm of HawkDock is composed of ATTRACT and HawkRank, which allows flexible protein–protein docking combined with MM/GBSA free energy decomposition of key protein residues involved in the interaction [23]. Prior to PPI, we selected chain-A of SARS-CoV-2 receptor binding domain as the rest of the chain had identical sequence as compared with the rest of the protein chains (B, C). Our strategy was to dock these selected proteins in the 319th to 591st number of amino acids within the chain-A of SARS-CoV-2 spike glycoprotein. These amino acids interact with the human ACE-2 receptor which facilitates their entry inside the brain, hence inflicting neuronal and neurovascular disruption in affected patients [26]. After successful docking process execution, the top 10 models were downloaded and analyzed for amino acid interaction between SARS-CoV-2 spike receptor and protein ligands and for the type of bond formed by each other. The hydrogen bonds in PPI holds the participating amino acid residues of one protein in bond formation with the other protein more vigorous than van der Waals forces [27]. By analyzing individual docked models, it was observed that, human neutralizing S230 light chain amino acid residues; serine 24 and serine 30 established H-bonds with the arginine 365, phenylalanine 388, tyrosine 267 of SARS-CoV-2 spike protein whereas valine 1 of S230 formed a H-bond with the tyrosine 312 having -22.94 kcal/mol of binding free energy (Supplementary Figure 1). On the other hand, ACE-2 formed van der Waal interaction with SARS-CoV-2 spike proteins with the binding free energy lower than mAb S230 as summarized in Table 1. DA protein created H-bond with leucine 390, alanine 313 and valine 266 with the binding energy of -26.35 kcal/mol (Supplementary Figure 2) whereas alpha 1 antitrypsin protein residue valine 107 created H-bond with arginine 271 (-27.6 kcal/mol binding free energy) of SARS-CoV-2 spike protein (Supplementary Figure 3) while both MBP- and S-18 peptide of human palate, lung and nasal epithelium clone protein (SPLUNC1) established only van der Waals interaction with the receptor protein having free energy of -21.8 and -40.34 kcal/mol (Supplementary Figures 4 & 5). The results are summarized in Table 1.

**Table 1.** Summary of protein–protein interaction results obtained from HawkDock.

Ligand (PDB ID)	Docked ligand-receptor (6VSB)	Model, docking score	Residues involved in H-Bonding (black) and van der Waal interaction (red)	BFE, kcal/mol
6NB6		Model 10, -4086.50	VALB1:TYRA312, SERB24: PHEA388, SERB30: ARGA365-TYR267	-22.94
4AWN		Model 01, -4132.90	TRPB269: VALA266, LYSB272: ALAA313, HISB392: LEUA390	-26.35
1ATU		Model 09, -4547.20	VALB107: ARGA271	-27.6
4N4X		Model 01, 4721.21	VALB454: ASNA361, ARGB455:HIEA392, ASPB456: TYRA360, LYSB457: LEUA363, GLNB458: ALAA393 GLYB466	-21.8
1R4L		Model-10,	VAL-B346: LEU-A367, THRB-316: TYRA-360, METB-314: TYRA-378, VALB-280: PROA-318, SERB-494: VAL-A321GLNB-287: ARGA-500, ALA-B283:VALA-527, LYSB-345: LYSA-573	-22.54

6NB6, chain L: Human neutralizing S230 light chain; 4AWN: Dornase-alfa; 1ATU: Alpha-1-antitrypsin 4N4X-MBP-fused human SPLUNC1; 6VSB, CHAIN A: COVID-19 spike glycoprotein; 1R4L: Inhibitor bound human angiotensin-converting enzyme-related carboxypeptidase (ACE-2). Residues involved in H-bonds (yellow) and VDW (red) ligand B: receptor A, binding free. A separate docking study was conducted for specific-S18 of SPLUNC1 peptide chain demonstrated a van der Waals interaction with 6VSB and a free binding energy of -40.34 (kcal/mol) (Supplementary Figure 5).

### Results of toxicity assessment

None of proteins/peptides: 6NB6, 4AWN, 1ATU, 4N4X and 1R4L demonstrate any kind of toxicity or allergic response *in silico*. Only SPLUNC1 demonstrated a 33.3% allergenic response in the *in silico* model. Apart from the specific S18 of SPLUNC1 peptide chain, they were nontoxic and nonallergenic. (Table 2) None of the proteins demonstrated resistance to the digestive enzymes. The purpose of conducting the resistance to digestive enzymes test was to check the fate of these inhalers as ultimately, they reach to the stomach and may cause adverse drug reactions. These inhalers were checked against the enzymes, such as trypsin, pepsin (pH > 2) and chymotrypsin. These inhalers are not able to protect themselves from these digestive enzymes, and are broken down in the stomach, and so cannot illicit unwanted physiological effects. The poor solubility of these inhalers may be due to the fact that all of them are protein/peptides and are hydrophobic in nature. The greater the aqueous solubility of these drugs, the greater will be the chance for these inhalers to illicit a response in the systemic circulation [28].

**Table 2.** Results of toxicity, allergenicity, digestion resistance and water solubility of protein/peptide-based inhalers.

Ligand (PDB ID)	Toxicity	Allergenicity	Digestion resistance	Water solubility
6NB6	Nontoxin	Nonallergen	No	Poor
4AWN	Nontoxin	Nonallergen	No	Poor
1ATU	Nontoxin	Nonallergen	No	Poor
4N4X	Nontoxin	33.3%	No	Poor
4NAX-S18	Nontoxin	Nonallergen	No	Poor

6NB6, chain L: Human neutralizing S230 light chain; 4AWN: Dornase-alfa; 1ATU: Alpha-1-antitrypsin 4N4X-MBP-fused human SPLUNC1; 4NAX-S18: Specific S18 of SPLUNC1 peptide chain.

## Discussion

SARS-CoV-2 spike glycoprotein is of great concern within the scientist community. These proteins can only reproduce by entering the cells. Blocking such entry might be of great interest in terms of treatment and that could be a way of preventing SARS-CoV-2 infection and flattening the disease curve [4]. We attempted to address this issue by analyzing a variety of protein/peptide-based inhalers/antimucolytic agents and previously utilized mAb (used in asthma) to observe their possible interaction with the SARS-CoV-2 spike protein. Both mAb and DA established H-bonds with spike protein employed by SARS-CoV-2 for attachment. However, in comparison with DA, the mAb and AAT showed considerable H-interaction with the SARS-CoV-2 spike protein. CF and chronic obstructive pulmonary disease (COPD) have high levels of DNA and actin in the lung lumen, which are discharged by necrotic neutrophils. The DNA population and actin alter mucus rheology as well as increase mucociliary clearance. Another way to boost mucociliary clearance in CF is to decrease mucus viscosity by cleaving extracellular DNA in infectious lungs. DA is a recombinant version of human DNase-1-protein which is used as a therapeutic moiety for CF [7]. Dnase1 cleaves extracellular DNA in the lung lumen, which results in reduced DNA length/concentration and decreased sputum viscosity [6]. This agent can also help in the treatment of COVID-19. SPLUNC1, on the other hand, does not demonstrate good interaction with the spike protein but does inhibit ENaC by inducing endocytosis incongruous to traditional ion channel antagonists which the block ENaC’s pore [8]. Regulation of ENaC fails because of SPLUNC1 in the CF lung, so, SPLUNC1-derived peptide acts in CF airways as an ENaC inhibitor. This regulates CF airway surface liquid hydration, boost mucociliary clearance and decrease infection, and inflammation [6]. These peptides are heat stable and with maximum binding efficiency to accomplish a greater contact area with their target proteins. S18-derived peptides do not readily cross the respiratory epithelium. They do not reach the kidney, where they might cause hyperkalemia because S18-derived peptides are protease-resistant as seen with small-molecule ENaC antagonists like amiloride. Chronic inhalation therapy results in local immunogenicity and irritation from the peptides involved, but it has been well established that SPLUNC1-derived peptides did not precipitate immunogenicity [6,8]. Our study highlights the toxic and antigenic profiles of the corresponding inhalers. Although the safety and efficacy of these inhalers, such as DA, SPLUNC1, AAT and mAbs were established already [16–19], we report the *in silico* antigenicity of SPLUNC1 protein while bearing in mind the potential side effects of mAbs [13]. Therefore, they could be utilized in the COVID-19 therapy and as an adjuvant.

Antibodies with therapeutic and diagnostic potential for SARS and MERS have already been identified [3,26]. The preparation of particular variants of antibodies that attach to SARS and MERS viruses are patented in 61 out of 99 registered agents. In 42 patents the S-protein in the viral infection exhibits an immune response. The scrutiny of patents associated with the establishment of therapeutic antibodies for SARS has been provided by Liu *et al.* [29]. These antibodies are 90% in opposition to S proteins along its RBD. Moreover, compared with chemical drugs, mAbs bind to multiple places on viral surfaces. This gives them an edge over chemical drugs. mAbs become attached to a wide variety of proteins with high affinity and specificity. After breakdown, the products are amino acids, so the resultant compounds are not toxic [6]. An exciting property of the mAbs is that they cling to their physical and immunological properties after the aerosolization, which implies that it is only a matter of time before mAb inhalation is exploited therapeutically. mAb and DA might synergistically clear the mucus, alleviate coughing and modulate the immune response toward the virion of the spike glycoprotein eventually disrupting the viral attachment. These inhalers could be used in in low-income countries where people cannot afford convalescent plasma therapy and where there is a shortage of inhalers/nebulizers.

On 24 March 2020, different pharmacists across the USA reported a shortage of metered-dose inhalers [30]. Unfortunately, despite the demand, the production of these inhalers has not kept pace. The problem was further aggravated by people stocking up on inhalers due to fear of the pandemic. However, despite their usefulness, these inhalers are not commonly available in pharmacies due to shortage of supplies. Pharmaceutical companies and health regulatory authorities in all the countries should ensure a proper supply of these inhalers.

## Conclusion

Because of the apparent devastating situation caused by COVID-19, computational studies can help in a quick drug repurposing analysis. mAb and DA demonstrated efficient binding with the spike protein of SARS-CoV-2. The protein/peptide-based inhalers are valuable assets due to their prolonged action, higher potency, lower systematic availability and minimum toxicity. Therapeutics for COVID-19 are in the infancy stage and no US FDA approved vaccine is available. During the pandemic, the use of protein/peptide-based inhalers along with other vaccines for SARS and MERS could mitigate the disease and serve as both therapeutic and prevention.

## Future perspective & recommendations

The general instructions for the use of these protein/peptide-based inhalers are:

- Follow all the manual instructions that are given by the manufacturers regarding the use and cleaning of the inhalers, compressors and nebulizers.
- These inhalers are usually present in solution form in plastic/glass ampoules.
- Air compressor is connected to a Jet nebulizer or eRapid™ nebulizer system to convert the drug (Pulmozyme^®^) into minutely small particles [31].
- The patients should breathe in the spray through a mouthpiece for about 10–15 min or until all the nebulizer cup is emptied.
- PARI BABY™ nebulizer must be used if the patient is unable to inhale or exhale.
- Never utilize a discolored or cloudy inhalation solution.
- Use it every day till your condition is ameliorated upon the direction of the physician.
- The frequency/dose of the drug should be calculated by the pharmacist on an individual basis according to the strength of the drug, severity of the symptoms, disease progression and body mass index.
- Precautions should be taken to avoid drug–drug and drug–food interactions.
- If you forget to take the medicine, do so when you remember, unless you are near the time of your following dose. In this case, do not take the dose you have missed, but return to the original schedule. Never double the doses.
- Administer one drug at a time and do not mix it with any other agents [31].
- The prescribers are advised not to switch between the inhaler types during the treatment unless otherwise required.

During the pandemic, certain areas of the world are experiencing shortages of the inhalers but it is not because of production problems. The spike in demand is due to patient overflow and the use of inhalers for respiratory difficulties experienced by the suspected and confirmed cases of the COVID-19. It is also due to the panic among the people and pharmacists who are ordering inhalers which are ultimately not needed in such high quantities. Other reasons according to the FDA are regulatory and logistical challenges in different countries. The manufacturers are not given incentives to produce economical inhalers [32]. This report provided some solutions to this issue including:

- Contracts with private companies to ensure a reliable supply of the inhalers.
- Incentives should be given to the manufacturers who provide a sustainable supply of good quality inhalers.
- Public sessions and seminars should be held to promote awareness of the impact of drug shortage on patients.

Different hospitals are moving away from the nebulizer use in order to avoid the spread of SARS-CoV2. So, the doctors are suggesting using these inhalers at home [30]. Virtual screening of the inhalers should be subjected to clinical trials before the issuance of the necessary medical recommendations. The *in silico* studies cannot completely replace the experimental studies, so we do not favor using these protein/peptide-based inhalers while first-line therapy options are available, nor do we advise readers to do so. But given the shortage of albuterol and other inhalers according to the FDA, the use of these peptide-based inhalers could be appropriate in the future.

**Summary points**

- Molecular docking analysis of protein/peptide-based inhalers revealed that the S230 light chain antibody and dornase-alfa demonstrated a strong affinity for SARS-CoV-2 spike protein.
- The binding free energy of the S230 light chain antibody and dornase-alfa were -22.94 and -26.35 kcal/mol indicating highly stable bonding with the spike receptor.
- Toxicity assessment of these inhalers suggest that they can be used as anti-spike attachment agents, except for SPLUNC1, which showed a 33.3% allergic reaction *in silico*.
- S230 light chain antibody, dornase-alfa and short-palate lung and nasal-epithelial clone-1-derived peptides are capable of impeding virus interaction with the human host cell.
