# Supplementary material for: In silico analysis of protein/peptide-based inhalers against SARS-CoV-2
Source: Future Virol. 2020 Oct 8:10.2217/fvl-2020-0119. doi: 10.2217/fvl-2020-0119 (PMC7543042; doi:10.2217/fvl-2020-0119)
Supplement: Supplementary file 1 [file fvl-2020-0119.s1.docx]

**SUPPLEMENTARY MATERIAL**


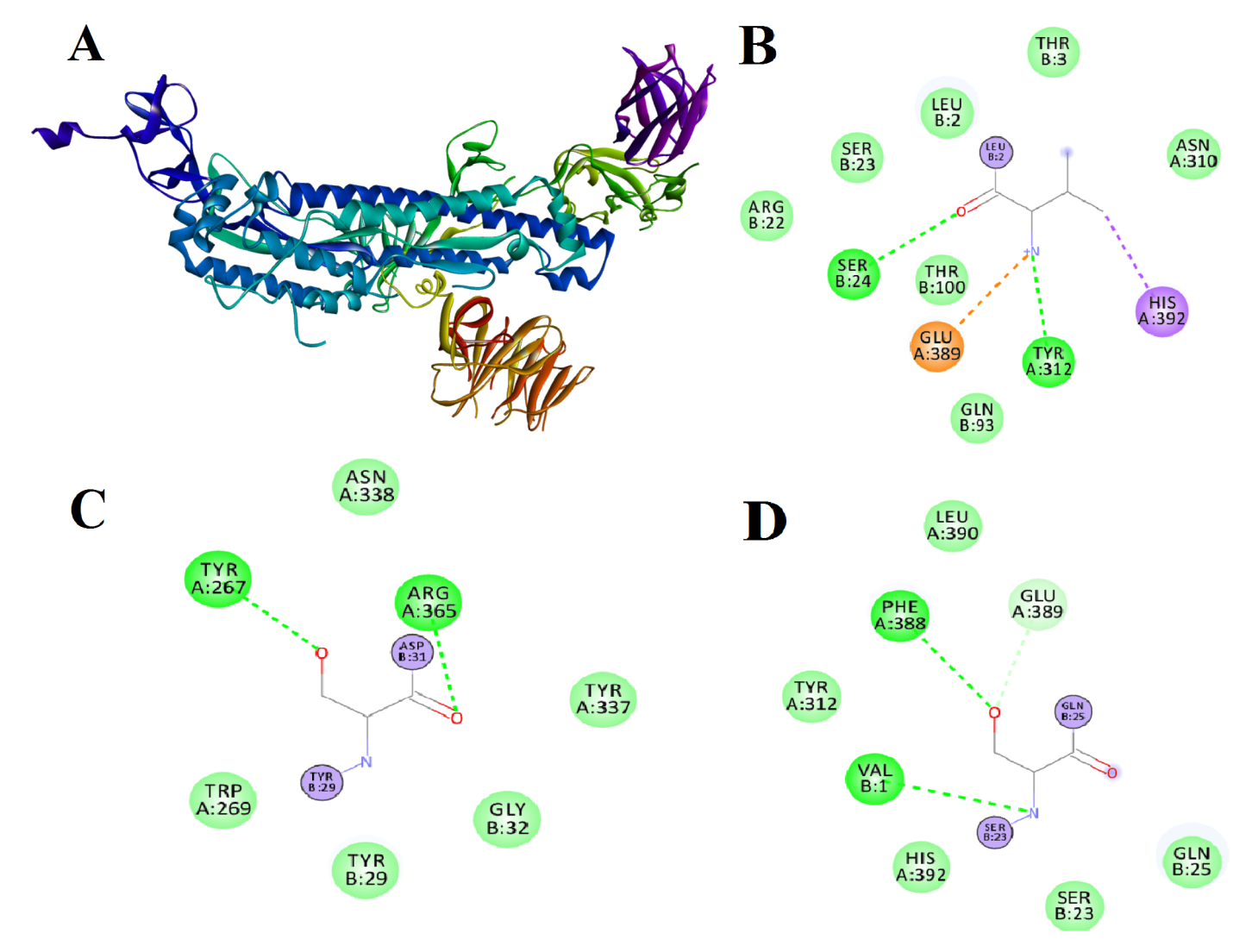


**Supplementary Figure 1**. Docking Interaction of Protein ligands with COVID Spike Protein receptor, 2D structural visualization of S230 Antibody amino acid residues: A) 3D docked structure of protein-protein interaction, B) Valine 1, van der Waals forces (light green), salt bridge (orange), H-bond (dark green), pi-bond (dark purple), covalent bond (light purple), C) Serine 24, van der Waals forces (light green), H-bond (dark green), covalent bond (light purple), D) Serine 30, van der Waals forces (light green), H-bond (dark green), covalent bond (light purple), with the amino acids of spike glycoprotein.


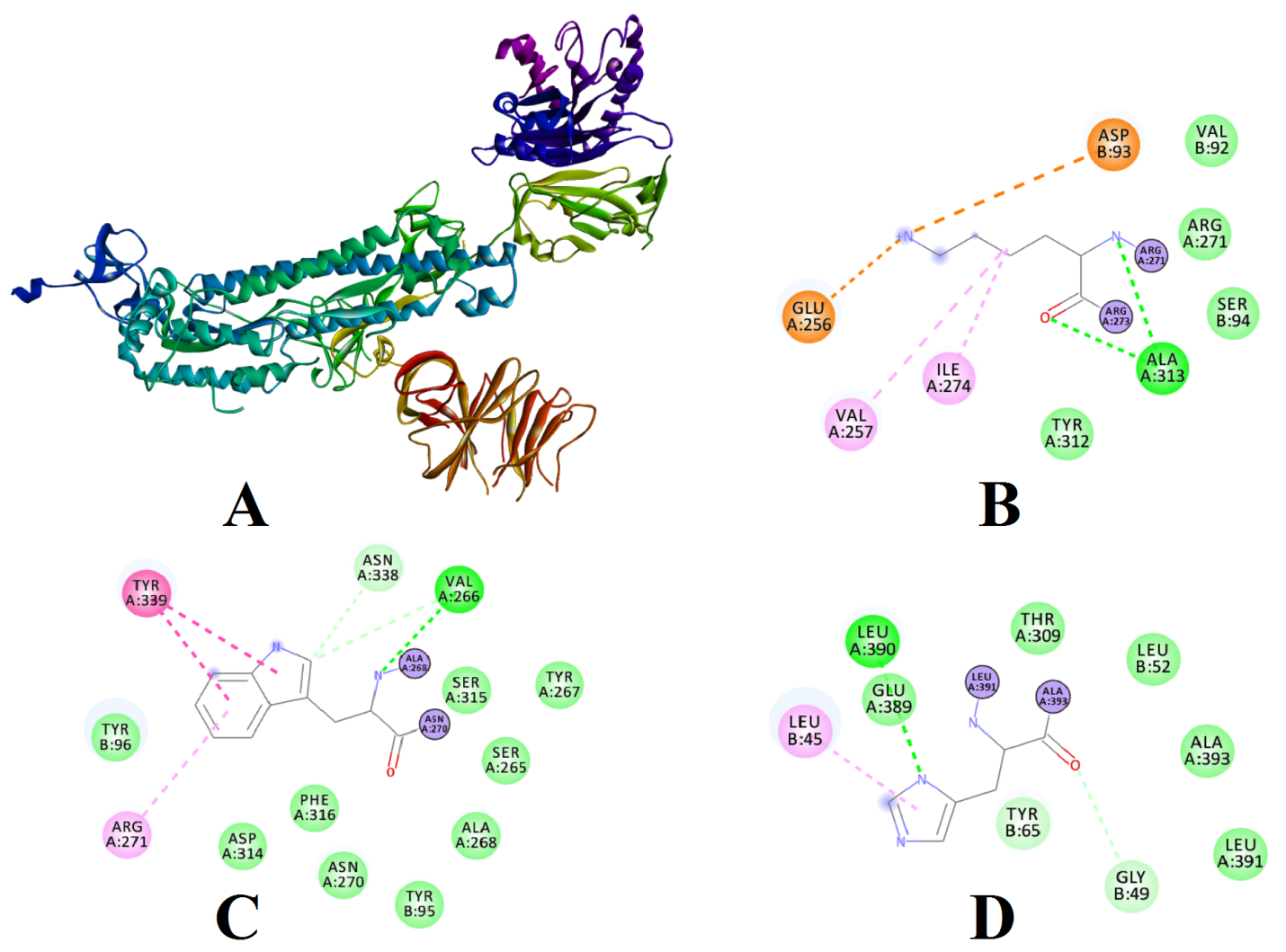


**Supplementary Figure 2**. A) 3D Interaction of Human DNAse-I with the COVID spike protein. 2D structural visualization of Human DNAse-I amino acid residues: B) Alanine 313, van der Waals forces (light green), H-bond (dark green), attractive charge (orange), allyl bond (pink), covalent bond (purple), C) Valine 266, van der Waals forces (light green), salt bridge, H-bond (dark green), pi-bond (pink), covalent bond (light purple), D) Glutanine 389, van der Waals forces (light green), H-bond (dark green), pi-bond (pink), covalent bond (light purple), with the amino acids of spike glycoprotein.


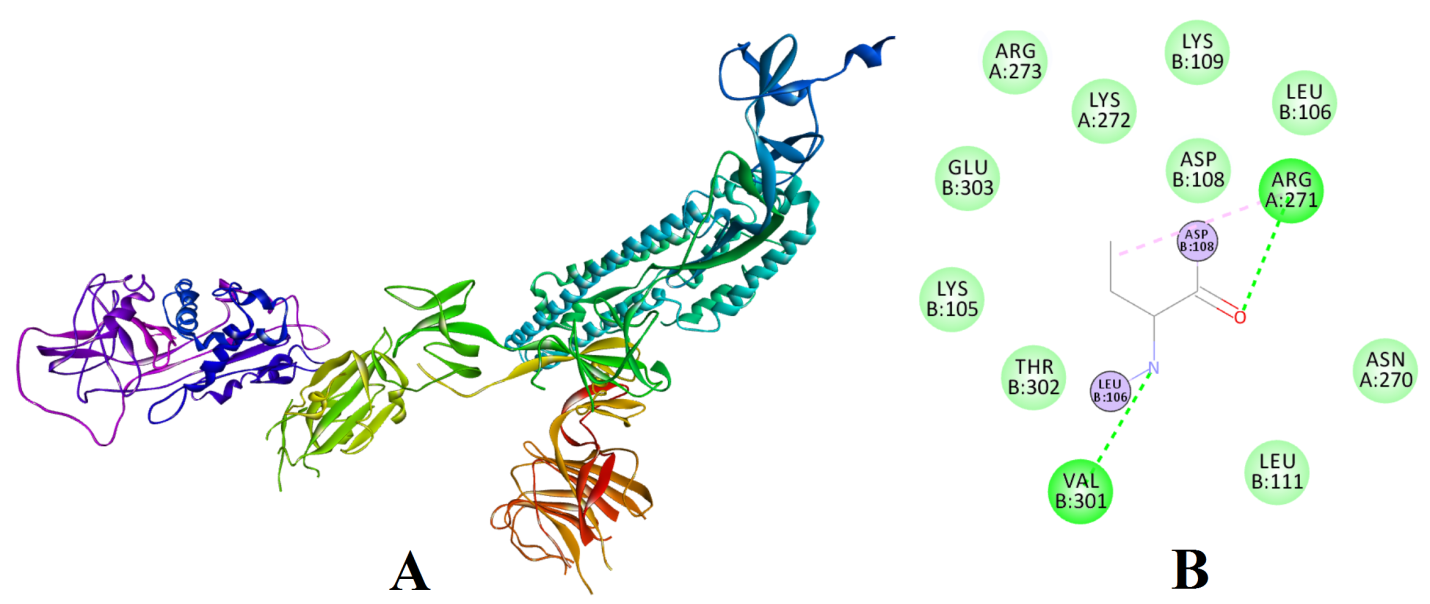


**Supplementary Figure 3**. Interaction of Alpha 1 Trypsin with the COVID spike protein. A) 3D Docking Interaction of Protein ligands with COVID Spike Protein receptor, B) 2D structural visualization of Alpha 1 Trypsin amino acid residues: Valine 301, van der Waals forces (light green), H-bond (dark green), allyl bond (pink), covalent bond (light purple) with the amino acids of spike glycoprotein.


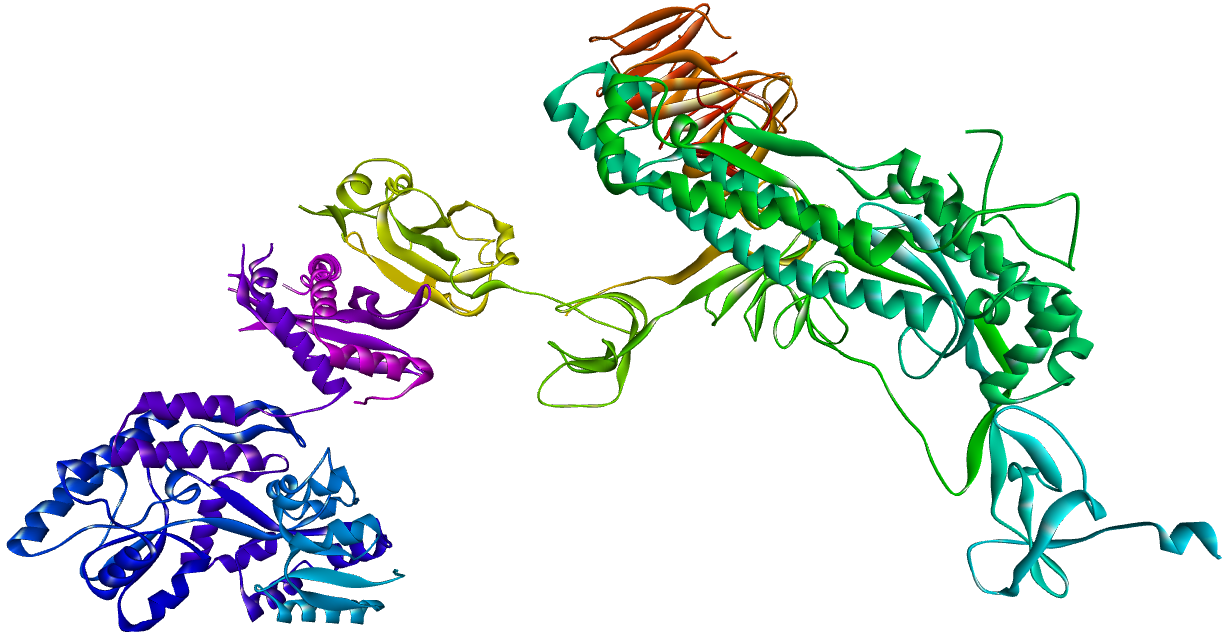


**Supplementary Figure 4.** Graphical representation of MBP-Human palate, lung and nasal epithelium clone protein docked with 6VSB


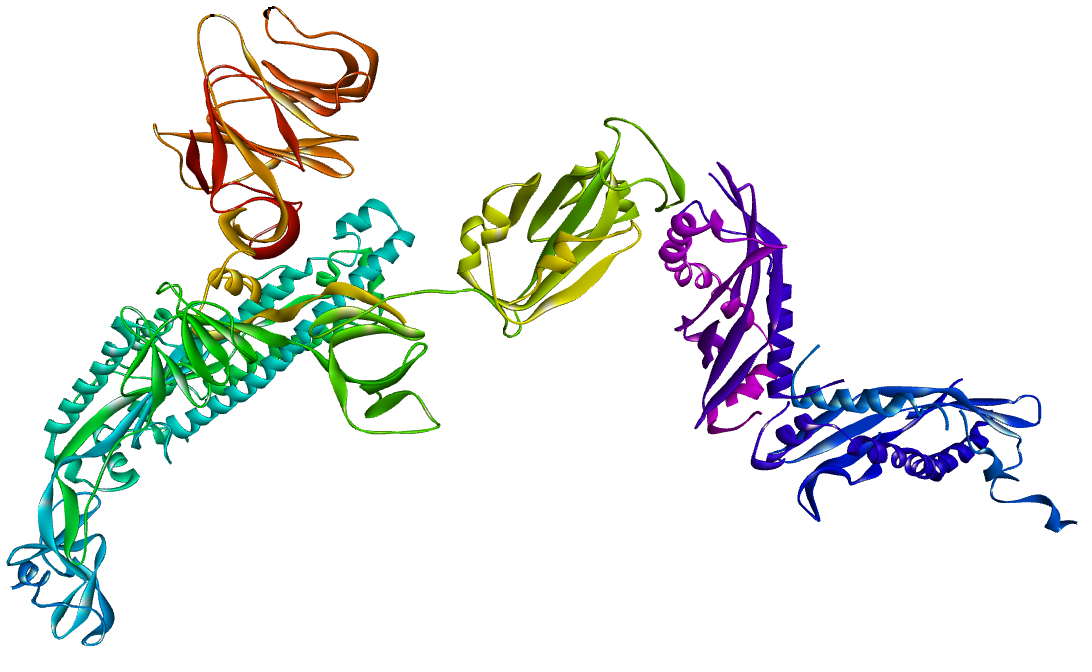


**Supplementary Figure 5.** Graphical representation of Human palate, lung and nasal epithelium clone-S18 peptide docked with 6VSB
